# Changing tracks: how different visual presentations of travel itineraries impact the choice between plane and train

**DOI:** 10.3389/fpsyg.2025.1588280

**Published:** 2025-07-21

**Authors:** Daniele Catarci, Lea Laasner Vogt, Ester Reijnen

**Affiliations:** School of Applied Psychology, ZHAW Zurich University of Applied Sciences, Zurich, Switzerland

**Keywords:** mode of transportation choice, travel itinerary presentation, travel duration, corporate policies, sustainability, plane/train travel, randomized controlled trial

## Abstract

Despite the negative impact flying has on the environment, people too often seem to choose the plane over the train because it supposedly “saves them time.” However, these perceived time savings are often overestimated, and in reality, can be significantly smaller because people have (deliberately or not) forgotten to consider the time costs incurred at the airport for security checks or baggage collection, for example. We therefore wondered whether this illusion of time savings could be prevented or reduced by visually highlighting the *total travel time*, thereby increasing the choice of train. In our first randomized online study (*N* = 614) on work-related travel scenarios, we were indeed able to show that presenting a comprehensive itinerary (visualizing the total travel time) instead of just the flight time (standard itinerary) increased train choice from 66 to 79%. A second study (*N* = 383) confirmed the robustness of this effect across different travel distances and price scenarios. Although our intervention worked, it may prove challenging to implement. A third study (*N* = 198) therefore examined an alternative intervention, a company guideline discouraging plane travel, by emphasizing both the environmental impact and the limited net time savings. The results showed a comparable increase in train choice. Overall, these results show that drawing attention to overlooked but critical attributes of decision-making, such as the actual total travel time, can serve as a powerful nudge for more sustainable travel choices.

## Introduction

One way to reduce climate changing CO_2_ emissions is to change our travel behavior, in particular to fly less. Although flights are “only” responsible for around 4% of global emissions (Klöwer et al., 2021), this figure is noteworthy when you consider that only a relatively small proportion of the world’s population, mainly from wealthy countries (e.g., Switzerland), flies. For example, only 11% of the total world population flew in 2018 (Gössling and Humpe, 2020). Hence, in Europe, for example, flights cause 14% of transport-related emissions, which is far more than trains (0.4%). In addition, aviation infrastructure also causes more emissions than that of rail transport (European Environment Agency, 2021). Therefore, especially in Europe, which is an area with dense air traffic, switching from plane to train could significantly reduce emissions.

However, literature shows that *behavioral change* is anything but easy (e.g., see Halpern, 2015; Sheeran and Webb, 2016; Thaler and Sunstein, 2021). In addition, the planned change involves a problem that can be illustrated using the scenario of “Ben,” who has to plan a *business-related* conference trip. While planning this trip (from Zurich to Florence), Ben carefully weighs up his travel options. One of them is a train journey through beautiful landscapes at a relatively low price. However, its duration of almost 6 h seems too long. After all, “time is money.” Ben therefore opts for the other option, a direct flight of just over an hour. On the day of the trip itself, however, when Ben finally arrives at the conference center in Florence, he realizes that the time it took him to get to Zurich airport, go through security, board the plane, wait for his luggage in Florence and take the bus to the city center, in addition to the flight itself, added up to a *total travel time* that was surprisingly close to that of the train journey. What happened to Ben is referred to as “time cost neglect” (Pan et al., 2024). But what annoys Ben most about his realization is that he should have been aware of these *additional time costs*; after all, it was not his first flight. This situation is not unlike US consumers not taking VAT (sales taxes) into account when buying groceries, as the prices of the goods on the shelves are shown without VAT. However, if those consumers are asked about the amount of tax to be added, they can state them fairly accurately for a whole range of products. Interestingly, when the VAT is stated in advance, it causes consumers to shop differently, and the demand for those goods decreases (Chetty et al., 2009). The question we now ask ourselves is whether the *explicit* addition of (implicitly) known missing information (i.e., the additional time cost) at the time of booking would have changed Ben’s (or anyone else’s) decision and thus led to less plane travel overall (and therefore less demand for this “good” or service)? Early indications that this could be the case were provided by Tversky and Kahneman’s (1981) famous “Asian disease” problem, or more precisely from its *critics*.

Kahneman and Tversky (1986) used this problem to illustrate that people do not always act according to the principles or basic assumptions formulated in “rational choice” theories (e.g., von Neumann and Morgenstern, 1953; Savage, 1954), such as *descriptive invariance*. Descriptive invariance assumes that the way in which identical (choice) options (e.g., yogurt A and yogurt B) are described linguistically or *framed* (e.g., whether yogurt A is 90% fat-free or contains 10% fat) should not influence people’s decisions (i.e., choosing yogurt A over yogurt B). They illustrated the violation of the principle by presenting participants with two versions of the problem (see Box 1), each giving participants a choice between a program with a “certain” outcome (A or C) and a program with a “risky” outcome (B or D).

A closer look at the problem reveals that the options or programs in each version have the same expected value (“EV”; 200 in version 1, 400 in version 2) and that the number of lives that can be saved in both versions is also the same [in version 1, 200 (out of a total of 600) people are saved; in version 2, 400 (out of a total of 600) people die].

Consequently, from a rational point of view, participants should therefore be indifferent in both versions regarding their choice between the two options or, if not, at least deviate in the same direction in their choice (i.e., always choose the “risky” option).

BOX 1 **“Asian disease” problem.**

Imagine that the United States is preparing for an outbreak of an unusual Asian disease, which is expected to kill 600 people. Two alternative programs to combat the disease have been proposed. Assume that the exact scientific estimates of the consequences of the programs are as follows:

[Version 1]

If Program A is adopted, 200 people will be saved.

If Program B is adopted, there is a 1/3 probability that 600 people will be saved and a 2/3 probability that no people will be saved.

It should be noted that half of the participants received version 1, while the other half received version 2.

[Version 2]

If Program C is adopted, 400 people will die.

If Program D is adopted, there is a 1/3 probability that nobody will die and a 2/3 probability that 600 people will die.

However, Tversky and Kahneman found that the way the options or programs were “framed” in the two versions—either as gaining/saving lives (gain framing) or losing lives (loss framing)—caused people to choose differently. That is, in the gain framing (version 1), 72% of participants chose program A, the *certain* option, while the opposite was observed in the loss framing (version 2): Here, 78% of participants chose program B, the *risky* option. Accordingly, gain framing makes people *risk-averse*, while loss framing makes people *risk-seeking*. Although these results are not consistent with rational choice theories, they can be well predicted by Kahneman and Tversky’s (1979) prospect theory, which uses a (neutral) reference point to categorize outcomes into gains or losses relative to that point. The resulting concave curve for gains or convex curve for losses results in the behavioral pattern observed above (e.g., risk aversion for gains). These “risky-choice framing” effects have not only been replicated many times (e.g., Kühberger, 1998; Levin et al., 1998) but have also been applied to *everyday* problems.

In this context (i.e., everyday problems) it was found that depending on how the problem is described, people can be encouraged to engage in *certain* (e.g., putting on sunscreen) or *risky* activities (e.g., going for a mammography or prostate screening), the problem at hand is described differently (whether the use of this method is morally/ethically right is another question, but needs to be considered). In this sense, for example, Detweiler et al. (1999) found that depending on whether the message on a flyer distributed at the beach about the use of sunscreen was described as a gain or a loss (e.g., “Protect yourself from the sun and you will help yourself stay healthy” or “Expose yourself to the sun and you risk getting sick,” p. 191), beachgoers intention (and also actual use) to use sunscreen with a sun protection factor (SPF) of 15 and higher was higher in the gain framing, especially if they had not previously planned to use it.

However, as authors such as Kühberger (1995); see also Mandel (2001, 2014), Kühberger and Tanner (2010), Tombu and Mandel (2015), and Gigerenzer (2018) have noted—and what is crucial for our work here—is that while in Tversky and Kahneman’s (1981) “Asian disease” problem in the risky options (programs B and D) all information is *explicitly* stated, this is not the case in the certain options (programs A and C). For example, program A says nothing about what happens to the remaining 400 lives. In this respect, however, it has been argued that people rightly assume (as in our case with air travel or VAT) that the remaining 400 people would lose their lives (Mandel, 2014), and accordingly it should be obvious by these simple mathematical calculations that, for example, program A and C are identical (called: proof by arithmetic argument, see, for example, Mandel, 2014). But, Kühberger (1995) has shown that if the missing information “400 people will die” (in program A) or “200 people will be saved” (in program C) is added to *certain* options, that is, made *explicit* (see also Mandel’s experiment 3), the effect demonstrated by Tversky and Kahneman (1981) disappears. There are many reasons why missing information could change the result. One of these is that, for example, the sentence “200 will be saved” is not understood as meaning that exactly 200 people will be saved, but rather that *at least* that many people will be saved (e.g., Mandel, 2014). Note that omitting part of the information in the risky options, as Reyna and Brainerd (1991) did (e.g., “2/3 probability that no people will be saved”) also changes the results (see also Kühberger and Tanner, 2010).

Hence, the *explicit mention of missing information* in the adapted versions of Tversky and Kahneman’s (1981) “Asian disease” problem influenced people’s decisions in a way that made them more similar across different versions (i.e., there was no preference for one program over another anymore). Let us come back to the plane-versus-train problem. It is important to note that in this regard both choice options (plane and train) presumably lie in the loss quadrant of prospect theory, as it is about “lost time,” and that they are both *certain* (here we momentarily overlook the possibility of delays and strikes for both modes of transport). The difference between the two options is that all time costs are listed for the train option, whereas this is not the case for the other option, the flight. In this case, only the time for the flight itself (approx. 1 h) is listed, but not the additional time costs incurred (approx. 4 h; missing information) for traveling to the airport, checking in, etc.

Now, the psychology of attention or, more specifically, the salience theory by Bordalo et al. (2012); see also Bordalo et al. (2022) may give us a clue as to why the explicit presentation of missing information as in Tversky and Kahneman’s (1981) “Asian disease” problem might change decisions. Consider thereby the following quote from Bordalo et al. (2012), p. 1255: “the decision maker evaluates lotteries^1^ by focusing on, and weighting more, their most salient states.” Applied to Ben’s situation, the most salient state is the available information of a short flight of about an hour. Consequently, this information is disproportionately weighted in his decision (see also Dertwinkel-Kalt et al., 2017 or the focus illusion by Kahneman et al., 2006). The presentation of the missing information could now lead to the information of the short flight becoming less salient, which reduces its weighting and thus also the decision in favor of the plane.

Although the *Asian disease* problem and its criticisms cannot be mapped *one-to-one* to our problem, we expect different decision-making behavior or choices (plane or train) depending on the *completeness* of the information provided. More specifically, the explicit mention of the (implicitly known) missing information should lead to the plane option becoming less salient, as its originally perceived advantage (short travel time) is reduced by the perception of the actual travel time, which should lead to the train being chosen more frequently.

In our case, we present the missing information not only verbally (i.e., with words), but primarily visually, for example, through a segmented visual timeline of the entire journey and the use of simple graphic elements (e.g., a train pictogram). This dual-modal approach is based on proven findings: visual information is better retained than purely verbal information [Paivio and Csapo, 1973; see also, for example, Osman and Thornton (2019) or Kühne et al. (2023) for the improved impact of labels as a supplement to purely textual nutritional information for healthier and more sustainable food choices]. Similar benefits (e.g., retention, understanding, decisions) can also be observed with pictograms. For example, the risk of reduced fitness to drive can be better assessed (and thus a more informed decision made) if the verbal information is supplemented by corresponding pictograms (Emich et al., 2014).

What is new about our work? First, testing the effect of displaying missing information in the appropriate decision context (i.e., switching from plane to train). Second, the mainly visual presentation of the missing information. Third, testing the effects through a real (randomized) experiment. And last but not least, testing the influence of other factors such as destination, travel distance and price structure in this context.

## Study 1

Study 1 examines, among other things, how the travel itinerary *presentation form* (standard vs. comprehensive) affects the choice of the mode of transport (plane or train). More specifically, a *standard itinerary* that only shows flight or train travel time is compared to a *comprehensive* itinerary that includes door-to-door travel time.

### Materials and methods

#### Participants

Six hundred and fourteen ZHAW Zurich University of Applied Sciences students aged between 18 and 51 (M_age_ = 24.6; SD_age_ = 4.9; 62.7% were female) took part in this *smartphone*-based online study. As an incentive, participants could either enter a lottery for an Apple iPad (which 87.8% did) or students from the ZHAW School of Applied Psychology (13.7%) could receive course credit instead (which 53.6% of them did). All participants gave their informed consent.

#### Stimulus material, procedure and design

**Figure 1:**
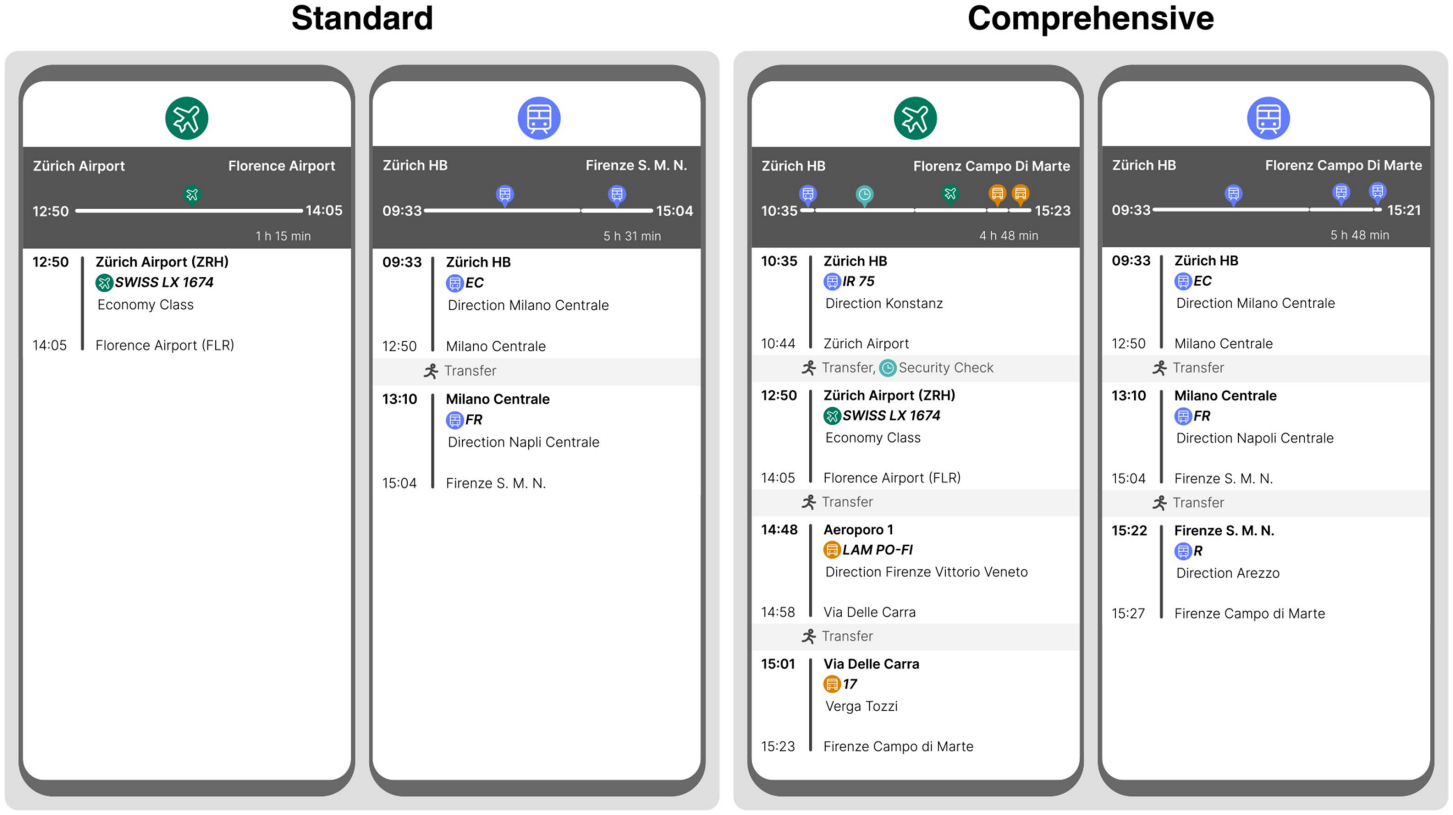
Stimulus material from Study 1, featuring itineraries for the trips from Zurich to Florence via train and plane, translated from German to English. In the standard condition, only the airport-to-airport flight and station-to-station train segments are shown (similar to most booking platforms). The comprehensive condition includes the entire journey to the hotel, incorporating travel to the airport, time for security checks, and local public transportation.

At the beginning of the study (implemented and run with the help of the Unipark software), participants were told (by means of a scenario) that they—as an employee of a medium-sized company—were to attend a conference abroad. They had to indicate which mode of transport (plane or train) they wanted to use for the main part of the journey, *starting in* Zurich HB (i.e., Zurich main station) and *ending* at the conference hall in Florence Campo di Marte (about 280 miles beeline). The travel itineraries for both modes of transport were presented to them visually, similar to a mobile booking app (see Figure 1). Depending on which (itinerary) *presentation form* condition participants were randomly assigned to, the journey was presented to them in the *standard* form (only the pure flight or train travel time) or in the *comprehensive* form (the complete door-to-door travel time, including, for example, the waiting time at the airport^2^; the two conditions form together the independent variable “presentation form”). In both presentation form conditions, participants had to indicate their mode of transport preference^3^ on a 0–100^4^ scale (0 = definitely by plane; 100 = definitely by train; dependent variable “preference”).

Thereafter, participants were asked to choose between two lunch options for the conference: one vegetarian and one meat based (dependent variable “menu choice”). This decision allowed for checking for any spillover or rebound effects (e.g., whether choosing the plane as mode of transport makes people more likely to choose the vegetarian menu). After these two tasks (mode of transport preference and menu choice), participants first had to rate both travel options (i.e., plane and train) in terms of the *specific travel factors*^5^ of convenience, time and reliability on a 0–100 scale (0 = very low; 100 = very high). They were then asked to rate how important the *general factors*^6^ of comfort, time effort, number of changes, arrival and departure time, sustainability and possibility to work were to them in relation to their chosen mode of transport (i.e., plane or train) on a scale of 1 to 5 (1 = not at all; 5 = very important). Finally, demographic data (age, gender, income and education) were collected to describe the study population.

### Results

Sixteen participants that needed more than 18 min to complete (see text footnote 6) the study were excluded from the analysis (2.5%). Statistical analyses were (also in Studies 2 and 3) performed with R software (version 4.2.3; RStudio Team, 2023).^7^ Wherever possible (i.e., in the case of continuous dependent variables), data were analyzed using *t*-tests or analyses of variance (ANOVAs), which are the so-called “bread and butter” methods of analysis in basic research (see Strasak et al., 2007); for details, see the relevant sections.

#### Mode of transport preference

**Figure 2:**
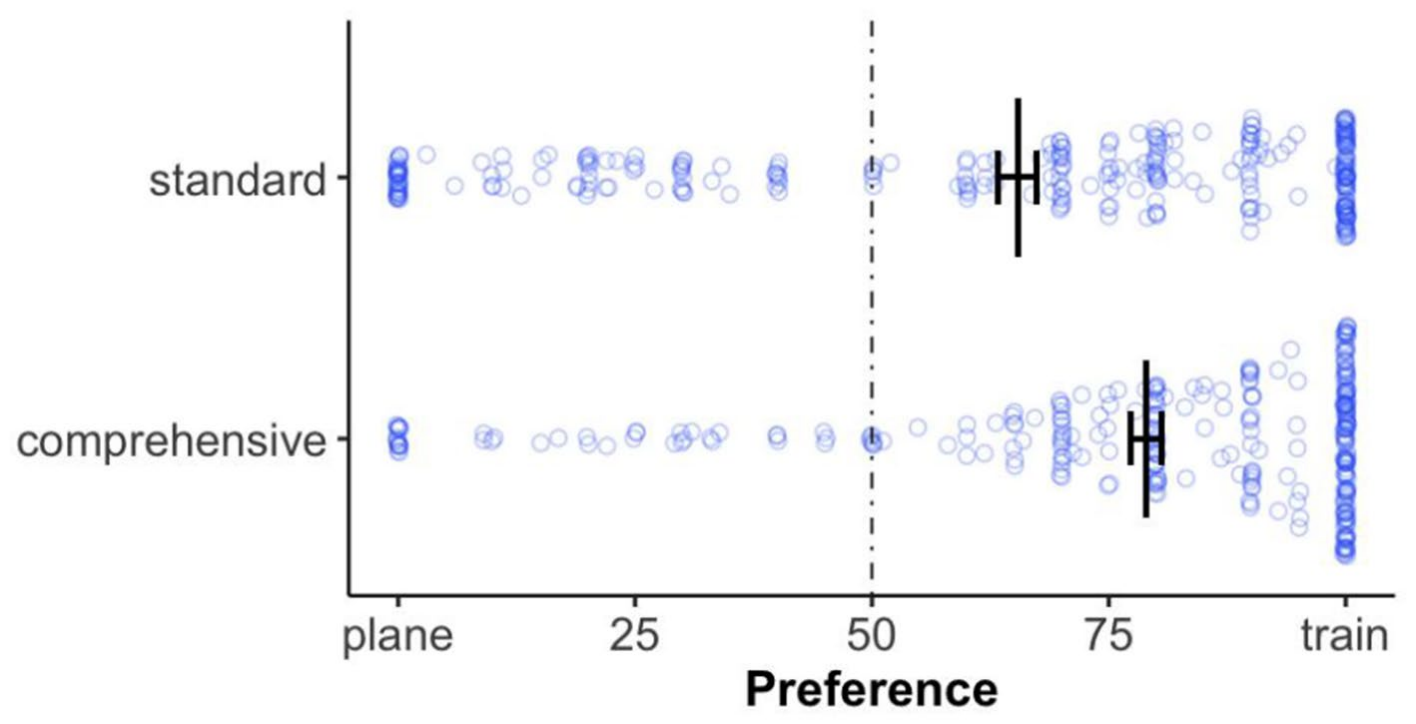
Mode of transport preferences by itinerary presentation, with each point representing an individual response. The dotted line indicates no preference for either option. Error bars represent standard errors of the mean.

A calculated unpaired *t*-test showed that participants preferred the train more often (about 20%) in the *comprehensive itinerary* than in the *standard itinerary*, *t*(612) = −5.23, *p* < 0.001, *d* = −0.42 (see Figure 2).

#### Decision time

**Figure 3:**
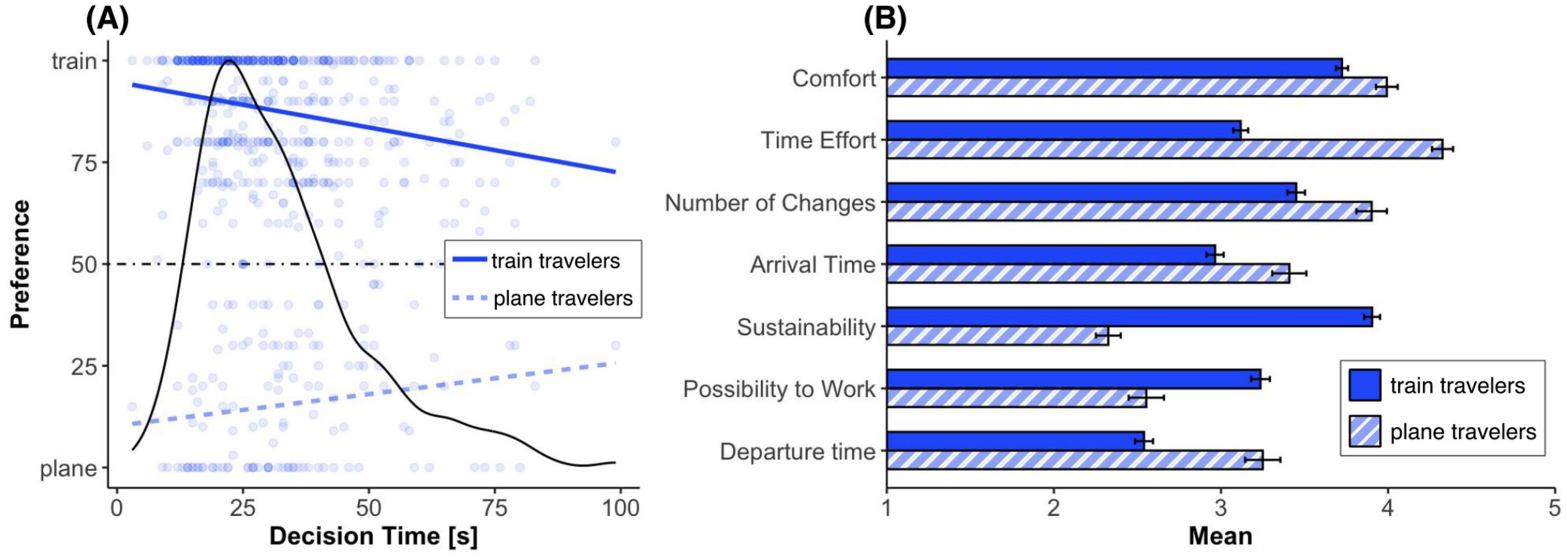
**(A)** Mode of transport preferences by decision time for train and plane travelers. The density plot shows the distribution of decision times, with each point representing an individual response. The dotted line indicates no preference for either option. **(B)** Mean importance scores for general decision factors for train and plane travelers, rated on a scale from 1 (not important at all) to 5 (very important). Factors are ordered by their overall mean. Error bars represent standard errors of the mean.

To investigate whether participants mode of transport preference (plane or train) was influenced by their decision time we conducted two one-way ANCOVAs (continuous factor: decision time in seconds^8^) on the dependent variable preference separately for plane travelers (*n* = 132; participants with <50% train preference) and for train travelers (*n* = 482; participants with ≥50% train preference). We found, for both plane and train travelers, a significant effect of decision time on preference [plane travelers: *F*(1, 128) = 4.56, *p* = 0.035, *η*_p_^2^ = 0.034; train travelers: *F*(1, 472) = 31.83, *p* < 0.001, *η*_p_^2^ = 0.063], although this effect was more pronounced for train travelers (see Figure 3A). Hence, with increasing decision time, preferences for both plane and train travelers shifted toward indifference (50%).

#### Specific travel factors

**Figure 4:**
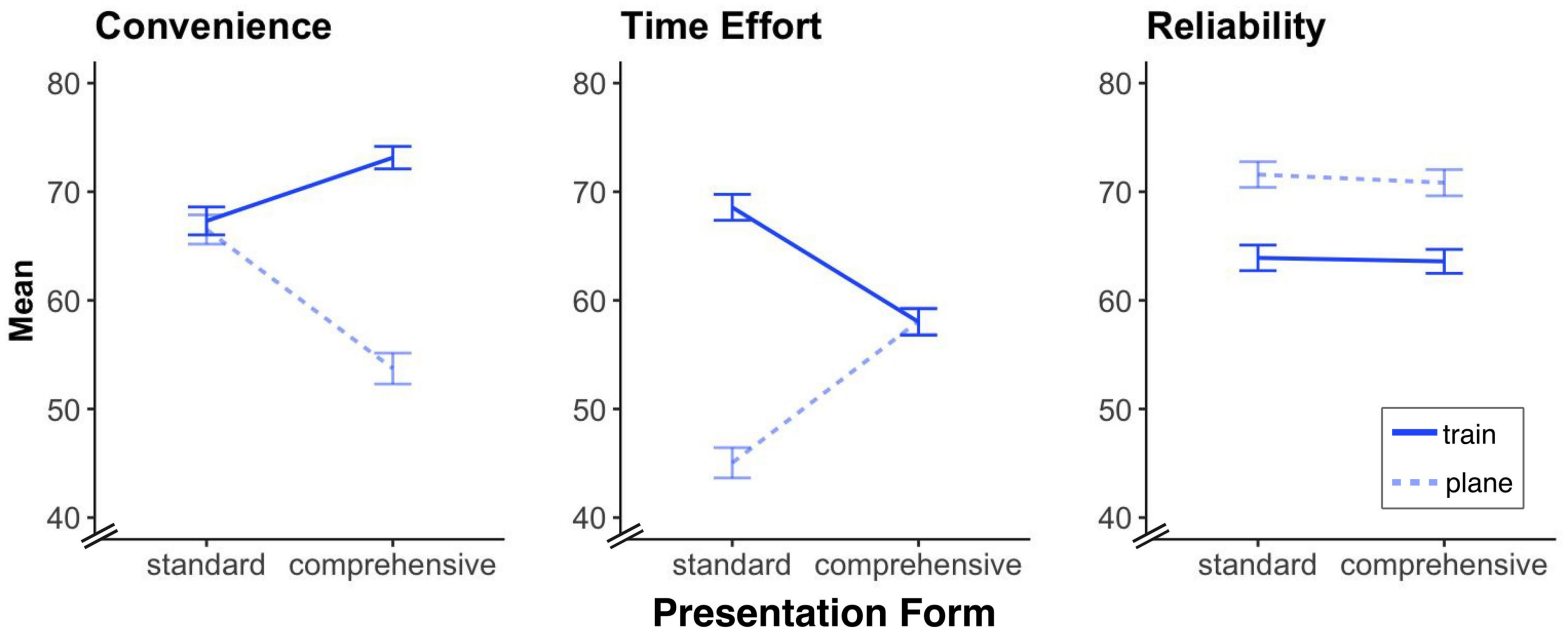
Specific travel factor ratings for the train and plane journeys under standard and comprehensive itinerary conditions. The figure shows mean ratings for convenience, time effort, and reliability on a scale from 0 (very low) to 100 (very high). Error bars represent standard errors of the mean.

How do participants (depending on the presentation form) rate traveling by plane compared to traveling by train, for example in terms of convenience? We conducted 2 (travel option: plane, train^9^; within-subject factor) × 2 (presentation form: standard, comprehensive; between-subject factor) ANOVAs separately on the dependent variable of participant’s responses to the specific travel factors of convenience, time effort, and reliability (see Figure 4).

##### Convenience

We found a significant main effect of travel option, *F*(1, 612) = 54.07, *p* < 0.001, *η*_p_^2^ = 0.081, indicating that the plane is viewed as a less convenient mode of transport than the train. We also found a significant main effect of presentation form, *F*(1, 612) = 8.76, *p* = 0.003, *η*_p_^2^ = 0.014, indicating that the standard itinerary presentation is viewed as more convenient than the comprehensive one. More importantly, however, is that we also found a significant travel option × presentation form interaction, *F*(1, 612) = 45.95, *p* < 0.001, *η*_p_^2^ = 0.070, indicating that the difference in convenience between plane and train was caused by the comprehensive itinerary presentation.

##### Time effort

We again found a significant main effect of travel option, *F*(1, 612) = 83.31, *p* < 0.001, *η*_p_^2^ = 0.120, indicating that traveling by plane is viewed as less time-consuming than traveling by train. In contrast to the previous findings, the main effect of presentation form was not significant, *F*(1, 612) = 1.02, *p* = 0.313. However, we again found a significant travel option × presentation form interaction, *F*(1, 612) = 84.37, *p* < 0.001, *η*_p_^2^ = 0.121, which indicates that the difference in time between plane and train was caused (this time) by the standard itinerary presentation and disappears when all trip-relevant information is displayed.

##### Reliability

We again found a significant main effect of travel option, *F*(1, 612) = 42.83, *p* < 0.001, *η*_p_^2^ = 0.065, with planes being viewed as more reliable than trains. However, neither the main effect of presentation form nor the travel option × presentation form interaction was significant (all *F* < 0.21, and all *p* >0.65).

#### General travel factors

How do participants [depending on the preferred mode of transport (plane traveler or train traveler)] rate general travel factors such as, for example, comfort (see Figure 3B)? The calculated unpaired *t*-tests (Bonferroni-corrected) showed that there is a significant difference between plane travelers and train travelers for each factor^10^ (comfort: *t*(612) = 3.49, *p* = 0.004, *d* = 0.34, time effort: *t*(612) = 13.27, *p* < 0.001, *d* = 1.30, number of changes: *t*(612) = 4.06, *p* < 0.001, *d* = 0.40, arrival time: *t*(612) = 3.99, *p* < 0.001, *d* = 0.39, sustainability: *t*(612) = −15.99, *p* < 0.001, *d* = −1.57, possibility to work: *t*(612) = −5.68, *p* < 0.001, *d* = −0.56, and departure time: *t*(612) = 6.08, *p* < 0.001, *d* = 0.60). However, time effort (travel time) seemed to be the most important factor for plane travelers, while sustainability was most important for train travelers. And it is precisely these two factors where the difference between plane and train travelers is greatest.

#### Menu choice

Finally, did participants choose the meat menu more or less frequently depending on their preferred mode of transport? A calculated Pearson’s chi-squared test (with Yates’ continuity correction) showed a significant association between “menu choice” (meat or vegetarian) and mode of transport (plane or train), *χ*^2^ (1, *n* = 610) = 4.51, *p* = 0.03, *V* = 0.01. While 40.15% of plane travelers chose the meat menu, the figure for train travelers was only 29.92%. Hence, a less sustainable mode of transport preference (i.e., plane) is therefore associated with the consumption of a less sustainable, meat-containing menu (and vice versa).

### Discussion

Study 1 shows that the presentation of comprehensive travel itineraries, which also include “hidden” time costs, increases the preference for train travel. However, it also became apparent that the relative disadvantage of the plane over the train diminished the more time the participants needed to make their decision. The observation that preferences can turn out differently depending on the duration of the “decision time” is also reflected in studies with *decoys*. In these studies, where there is a choice between two options (e.g., A and B), a third option (i.e., a decoy) is added to encourage the choice of a particular option (e.g., A). Now, research has shown that the effect of the decoy, a type of nudge (see Thaler and Sunstein, 2021), disappears when, for example, the decision maker is given enough time to make a deliberate cognitive decision (Gaudeul and Crosetto, 2019). In considering that our travel itinerary manipulation (making all travel time information explicit) is also a kind of nudge [probably a type-2 nudge; see Grüne-Yanoff (2025) or Hansen and Jespersen (2013) for a discussion of the different types of nudges], it should show the same effect, which was the case. In addition, it was found that while the time required (travel time) and the departure time were important factors for plane travelers in the decision, for train travelers it was sustainability and the opportunity to work. Finally, what is the link between the preferred mode of transport and the choice of menu? Do we observe “moral licensing,” that is, the phenomenon that a person who has made a decision that is perceived as morally good feels justified in subsequently making a less responsible decision (Merritt et al., 2010)? Or, in the context of our study, would participants who chose the environmentally friendly train option subsequently choose the less environmentally friendly menu (meat)? Our results show the opposite of that which would be expected under moral licensing: participants who preferred the train were more likely to also choose the vegetarian menu. This suggests that participants were more likely to act on an underlying attitude toward the environment, which in general leads to the choice of more sustainable services or products.

## Study 2

In addition to repeating the key results from Study 1, the aim of Study 2 was to examine selected results from Study 1 in more detail and to expand on the results. As can be seen in Figure 3B of Study 1, there is a greater difference between plane and train travelers in the factor “time effort (travel time)” than in the “number of changes.” This could indicate that these two factors do not cover the same underlying concept. Therefore, we also manipulate the number of changes in Study 2. Furthermore, we are interested in whether we can observe the same results as in Study 1 in relation to a longer trip. Last but not least, we are interested in how certain price structures (absolute vs. relative differences; see Kahneman and Tversky, 1984) affect the choice between plane and train.

### Materials and methods

#### Participants

Three hundred eighty-three ZHAW Zurich University of Applied Sciences students aged 18 to 49 (M_age_ = 25.2; SD_age_ = 4.9; 66.3% were female) took part in this *smartphone*-based online study. As an incentive, participants could either enter a lottery for an Apple iPad (which 92.2% did) or students from the ZHAW School of Applied Psychology (8.6%) could receive course credit instead (which 54.5% of them did). All participants gave their informed consent.

#### Stimulus material, procedure and design

**Figure 5:**
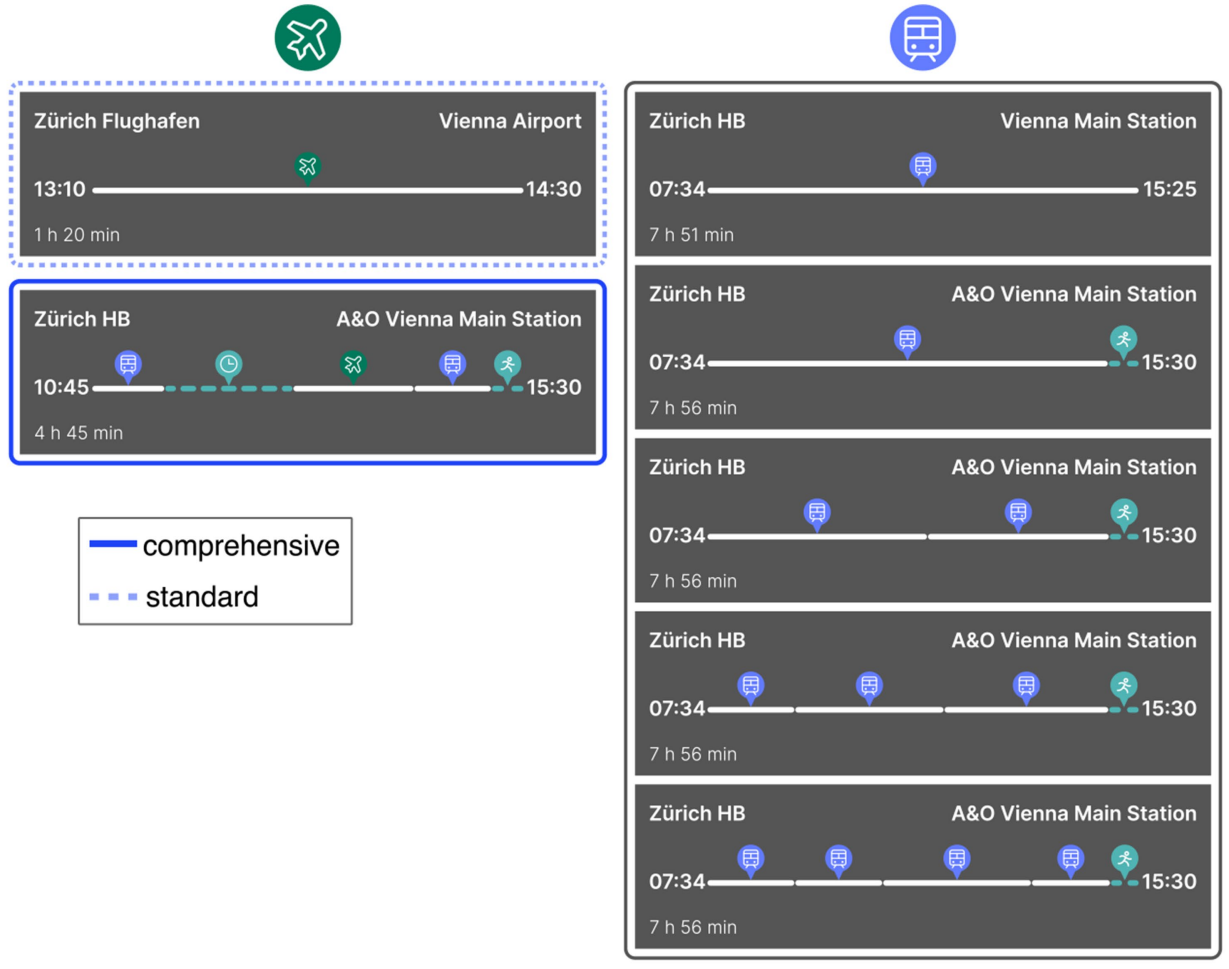
Exemplary stimulus material from Study 2 for the Vienna trip. Plane itineraries are displayed on the left, while train itineraries are on the right, featuring a single standard version at the top, and multiple comprehensive versions with 0–3 changes.

The stimulus material, procedure, etc. were similar to those in Study 1, such as the manipulation of the *presentation form* (standard, comprehensive; *between-subject* variable). However, this study introduced several key extensions. The following (between- or within-subject) variables were also manipulated:

1. The number of train changes (from 0 to 3 times) in the comprehensive travel itinerary (independent *between-subject* variable: changes; see Figure 5).
2. The destination, by adding to the relatively short route from Zurich HB (i.e., Zurich main railway station) to Cologne (main railway station; beeline: about 260 miles) a longer route from Zurich HB to Vienna (main railway station beeline: about 370 miles—see Table 1 for travel durations; independent *within-subject* variable: destination).
3. The price information, in that participants had to make the same choice twice, once on the condition that the company would pay for the trip and once on the condition that they would have to pay for the trip themselves. For the latter choice, the corresponding prices were communicated. Since we are primarily interested in how companies can influence employees’ travel choices, the first choice was always the one without price information. This manipulation [independent within-subject variable price (with/without)] allows us to investigate whether employees would choose differently if they had to pay the costs out of their own pocket. Furthermore, with regard to the “with price” condition, the following within-subject variables were manipulated: The relative price difference between the plane and train was set to be either “small” or “large” for a participant, and this was applied consistently to both destinations. The absolute price level (low and high) was also manipulated. This was varied for each participant in a counterbalanced manner, such that one destination was randomly set to a high price level and the other to a low level. The details of the price structures are shown in Table 2. Note that the train price for these routes—in line with reality—was always the lower one.

**Table 1:** Travel durations.

Destination	For the entire travel		For the main part	
	Plane	Train	Plane	Train
Cologne	4 h 25 min	5 h 20 min	1 h 10 min	5 h 15 min
Vienna	4 h 45 min	7 h 56 min	1 h 20 min	7 h 51 min

**Table 2:** Prices across the different conditions.

Price structure		Ticket price (in CHF)	
Price level	Price difference	Plane	Train
Low	Small (−10%)	80	72
	Large (−50%)	80	40
High	Small (−10%)	210	189
	Large (−50%)	210	105

Further, reliability, price and simplicity were added to the more general factors already tested above.^11^ Finally, participants were asked to rate their mobility habits (e.g., use of car, plane, cruise ship) as well as their beliefs about the environment (captured with the Environmental Portrait Value Questionnaire, or E-PVQ). However, this data or its results are not included in this manuscript.

### Results

Thirty participants that needed less than 4 or more than 30 min to complete the study were excluded from the analysis (which represented 7.3% of the total number of participants).

#### Mode of transport preference

##### Destination

**Figure 6:**
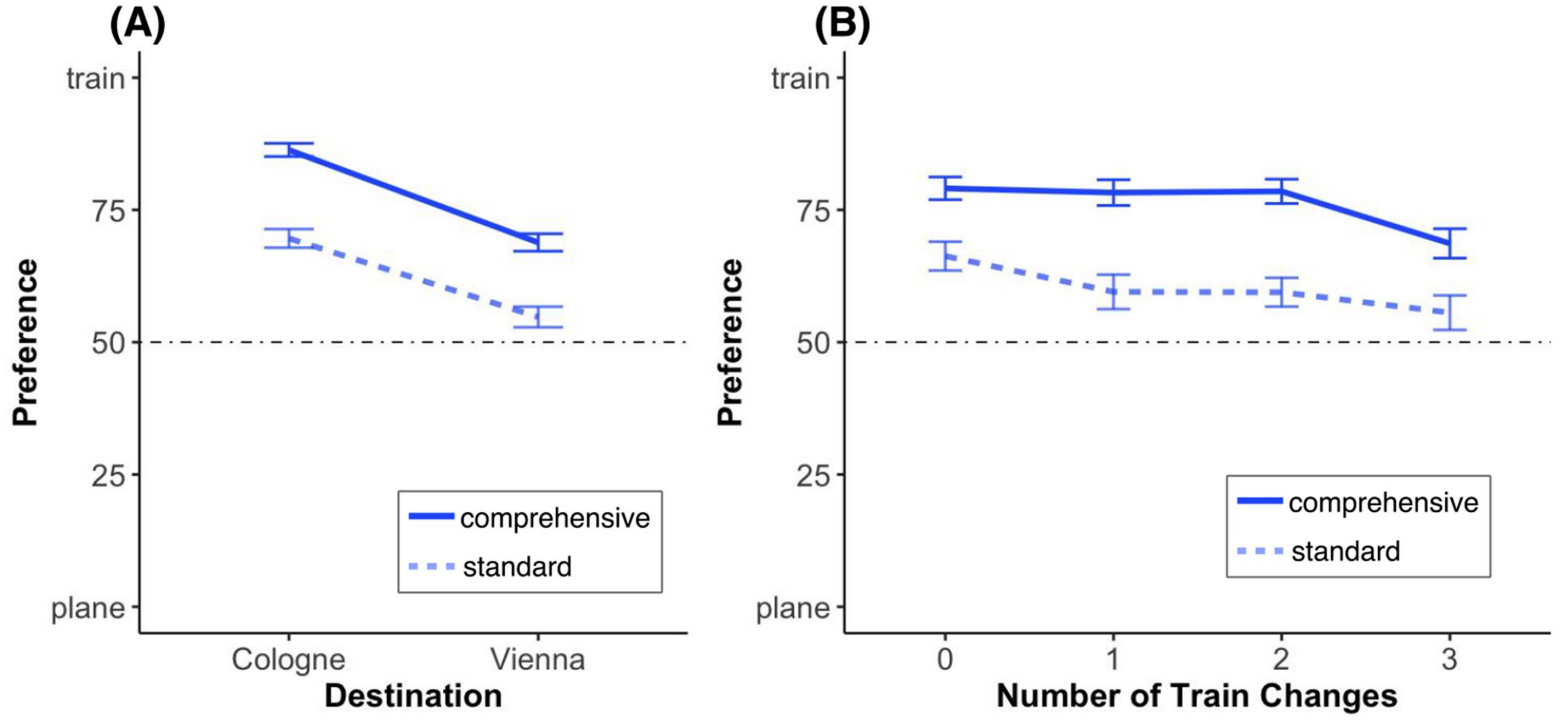
Mode of transport preferences by plane itinerary presentation and **(A)** destination or **(B)** number of train changes. The dotted lines indicate no preference for either option. Error bars represent standard errors of the mean.

We conducted a 2 (presentation form: standard, comprehensive; between-subject) × 2 (destination: Cologne [closer], Vienna [further]; within-subject) ANOVA^12^ with preference as the dependent variable. We found a significant main effect of presentation form, *F*(1, 381) = 29.37, *p* < 0.001, *η*_p_^2^ = 0.072, with a 15.4% higher preference for the train in the itinerary that was presented comprehensively. We also found a significant main effect of destination, *F*(1, 381) = 148.15, *p* < 0.001, *η*_p_^2^ = 0.28, with a 16.2% higher preference for the train in closer destination (Cologne). However, we found no significant presentation form × destination interaction, *F*(1, 381) = 0.98, *p* = 0.323, suggesting that the effect of itinerary presentation form on mode of transport preference was the same across destinations (see Figure 6A).

##### Number of train changes

To investigate the role of the number of train changes in more detail, we conducted a 2 (presentation form: standard, comprehensive; between-subject) × 4 (number of changes: 0–3; between-subject) ANOVA^13^ with preference as the dependent variable. We again found a significant main effect of presentation form, *F*(1, 305) = 8.67, *p* = 0.003, *η*_p_^2^ = 0.028. In addition, we again found a significant main effect of the number of changes, *F*(1, 305) = 4.69, *p* = 0.031, *η*_p_^2^ = 0.015, with participants being less inclined to take the train if they had to change trains more often. However, we again found no presentation form × number of changes interaction, *F*(1, 305) = 0.006, *p* = 0.938, suggesting that the effect of presentation form on mode of transport preference is the same over the number of changes (see Figure 6B).

##### Decision time

**Figure 7:**
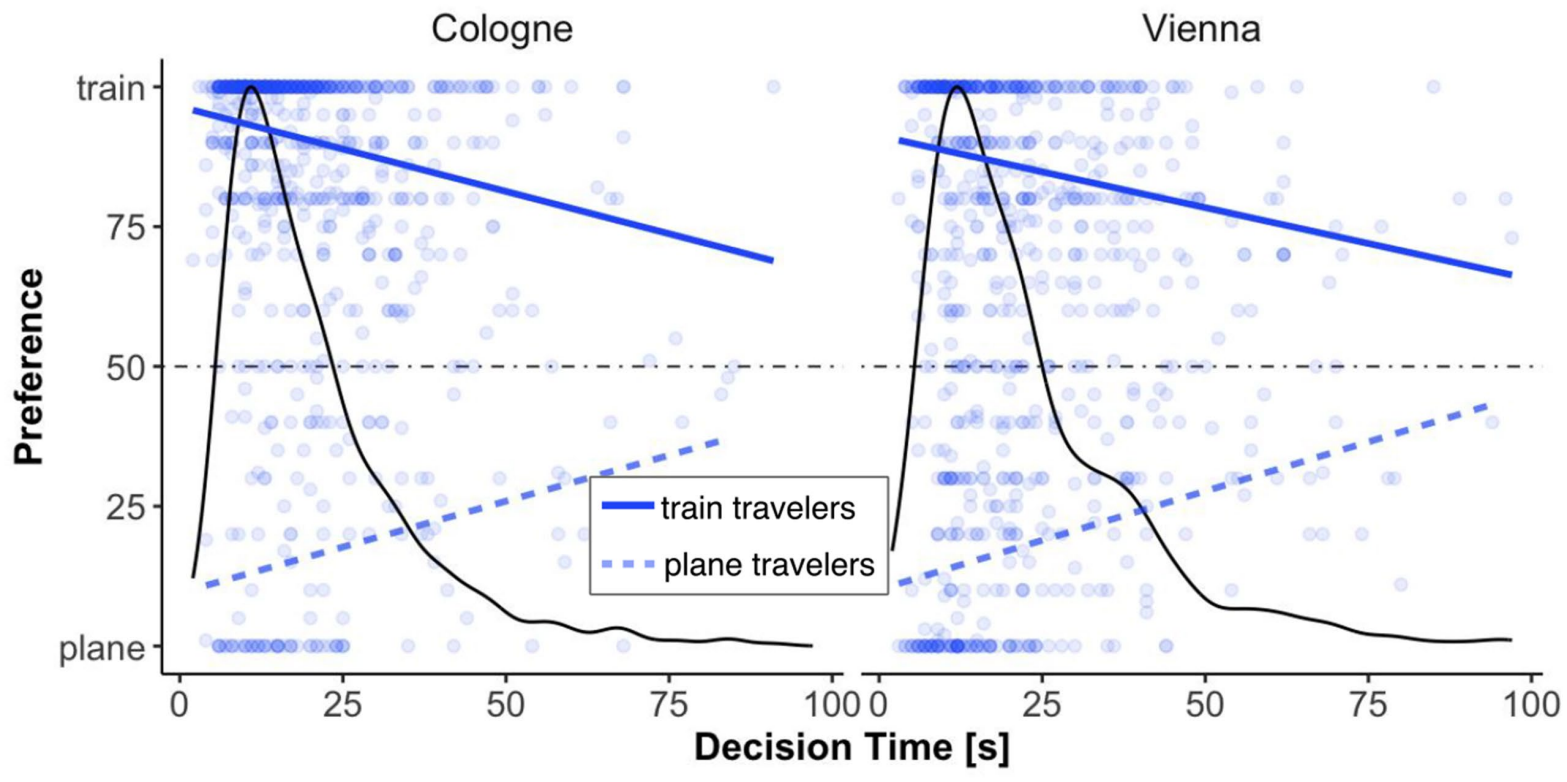
Mode of transport preferences by decision time and destination for train and plane travelers. The density plot shows the distribution of decision times, with preference trends for plane and train travelers for Vienna and Cologne. Each point represents an individual response.

We again investigated whether participants’ mode of transport preference was influences by their decision time (the specifics are the same as in Study 1). Independent of the destination (Cologne: short, Vienna: long), decision time affected both plane and train travelers (Cologne: plane travelers [*F*(1, 123) = 13.85, *p* < 0.001, *η*_p_^2^ = 0.101], train travelers [*F*(1, 620) = 53.91, *p* < 0.001, *η*_p_^2^ = 0.080]; Vienna: plane travelers [*F*(1, 257) = 37.96, *p* < 0.001, *η*_p_^2^ = 0.129], train travelers [*F*(1, 492) = 31.89, *p* < 0.001, *η*_p_^2^ = 0.061], see Figure 7). Hence, as in Study 1, with increasing decision time, preferences for both plane and train (independent of destination) travelers shifted toward indifference (50%).

##### Price

**Figure 8:**
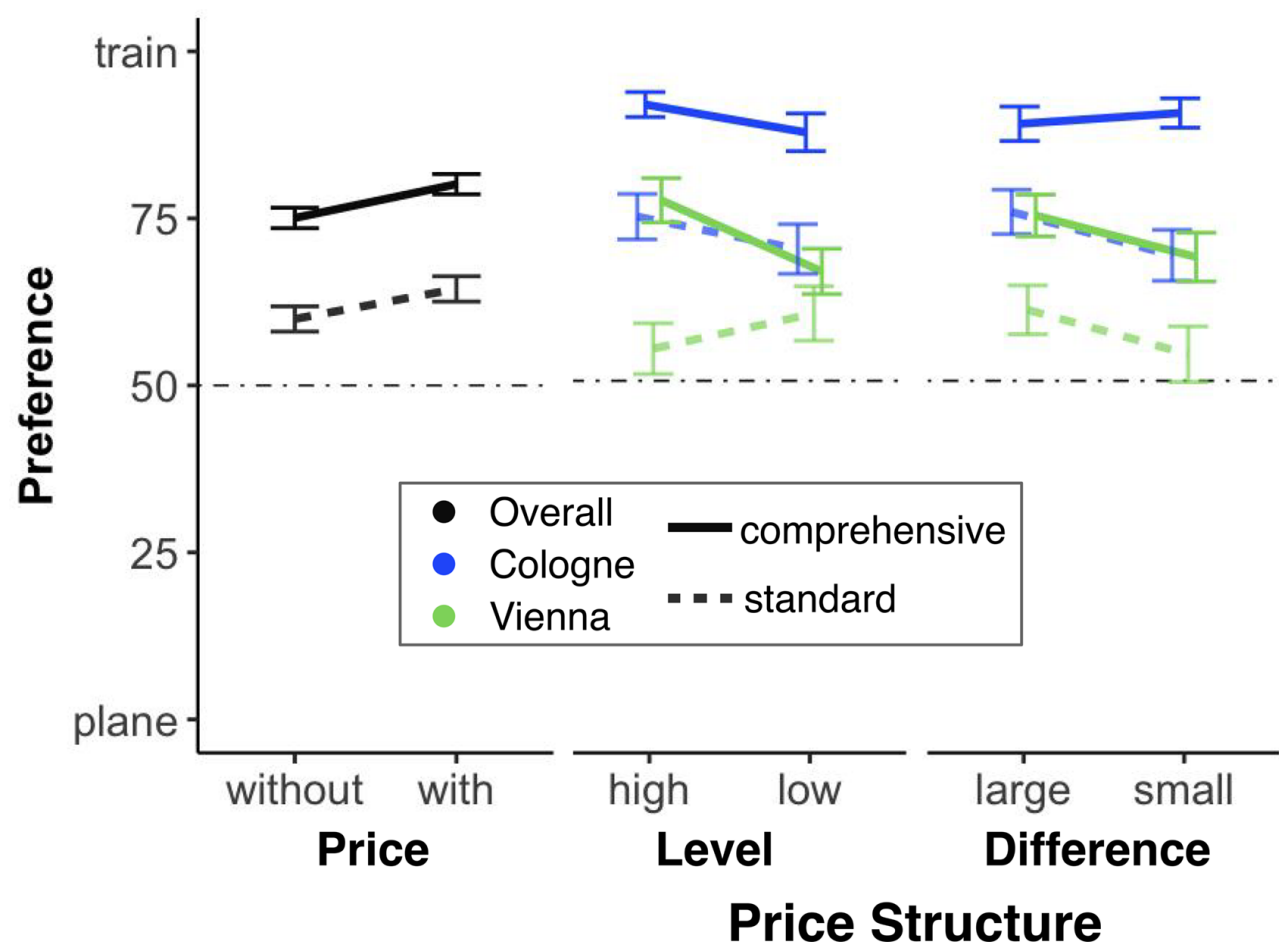
Mode of transport preferences by plane itinerary presentation and price structure. The dotted line indicates no preference for either option. Error bars represent standard errors of the mean.

First, we investigated whether the indication of price (as opposed to no indication of price) influences the mode of transport preference at all. Therefore, we conducted a 2 (presentation form: standard, comprehensive; between-subject) × 2 (price: without, with; within-subject) ANOVA with preference as the dependent variable. We found significant main effects for both presentation form, *F*(1, 381) = 29.37, *p* < 0.001, *η*_p_^2^ = 0.072, and price, *F*(1, 381) = 36.45, *p* < 0.001, *η*_p_^2^ = 0.087. However, we did not find a significant presentation form × price interaction, *F*(1, 381) = 0.12, *p* = 0.727. This means that although participants are more likely to choose the (cheaper) train option when they must pay for the tickets themselves, the observed effect of itinerary presentation form does not differ from that observed when the employer pays for the tickets (see Figure 8). Second, in the condition where the price was indicated, we investigated how the specific price structure (i.e., variation in price level and price difference) influenced the mode of transport preference (for Cologne and Vienna separately). Therefore, we calculated a 2 (presentation form: standard, comprehensive; between-subject) × 2 (price level: low, high; between-subject) × 2 (price difference: small, large; between-subject) ANOVA with preference as the dependent variable, separately for each destination (Cologne, Vienna). For Cologne, we only found a significant main effect of presentation form, *F*(1, 375) = 32.19, *p* < 0.001, *η*_p_^2^ = 0.079, but not for price difference, *F*(1, 375) = 0.65, *p* = 0.42, or price level, *F*(1, 375) = 2.25, *p* = 0.13. Furthermore, none of the interactions (2-fold or higher) were significant (all *F* ≤ 1.89, *p* ≥ 0.17; see Figure 8). For Vienna, we found a significant main effect of presentation form, *F*(1, 375) = 15.28, *p* < 0.001, *η*_p_^2^ = 0.039. All the other main effects were not significant, as were all interactions (2-fold or higher; all *F* ≤ 3.24, *p* ≥ 0.073), except for the presentation form × price level interaction, *F*(1, 375) = 4.58, *p* = 0.033, *η*_p_^2^ = 0.012 (see Figure 8). Looking more closely at this interaction, we found no significant effect of presentation form, *F*(1, 180) = 1.48, *p* = 0.23, when the price level was low, while the same effect was significant, *F*(1, 195) = 19.32, *p* < 0.001, *η*_p_^2^ = 0.090, when the price level was high.

#### General travel factors

Again, we investigated how participants [depending on the preferred mode of transport (plane or train) and destination (Cologne or Vienna)] rated general travel factors such as, for example, comfort (see Supplementary Figure 1). The calculated unpaired *t*-tests (Bonferroni-corrected; see Supplementary Table 1) again showed that time effort (travel time) seemed to be the most important factor for plane travelers, while sustainability was the most important factor for train travelers.

### Discussion

We demonstrated that the effect of presentation form observed in Study 1 is robust, independent of distance (destination: Cologne or Vienna) or the number of changes. This effect persisted when prices were included; however, the price conditions did not align with Kahneman and Tversky’s (1984) predictions. There was no overall effect of relative price differences, and while itinerary presentation remained consistent across most price conditions, there were exceptions. Specifically, itinerary presentation form had no impact when the flight was very cheap. This suggests that when perceived time savings from flying exceed a certain threshold and the plane option is clearly the better deal, comprehensive itineraries do not increase the likelihood of choosing the train.

## Study 3

Studies 1 and 2 showed that presenting a trip through a *comprehensive itinerary* encouraged participants to make more sustainable travel choices (i.e., preferring the train more often). By implementing *comprehensive itineraries* in existing booking platforms (e.g., booking.com), these platforms could contribute to more sustainable travel. Not wanting to rely solely on booking platforms to implement this intervention, the question arises whether there are other ways of encouraging participants—employees of companies—to choose the train more often. In Study 3, we explore whether introducing a company guideline which indicates that traveling by plane does not save (much) time would be an effective option.

### Materials and methods

#### Participants

One hundred and ninety-eight ZHAW Zurich University of Applied Sciences students aged 20 to 49 (M_age_ = 26.1; SD_age_ = 5.29; 68.7% were female) took part in this *smartphone*-based online study. As an incentive, participants could either enter a lottery for four 100 CHF gift cards (which 91.9% did) or students from the ZHAW School of Applied Psychology (16.2%) could receive course credit instead (which 28.1% of them did). All participants gave their informed consent.

#### Stimulus material, procedure and design

**Figure 9:**
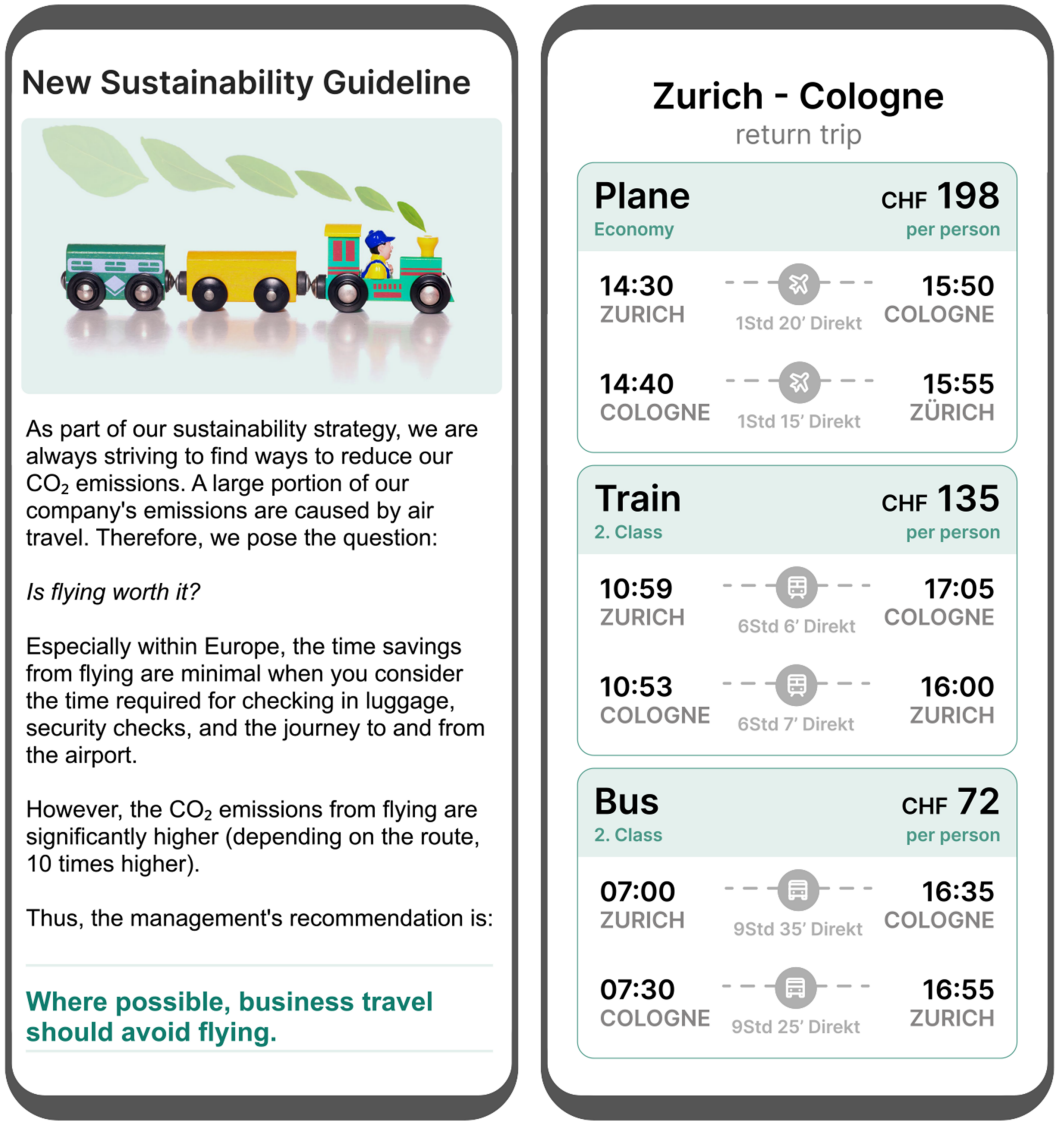
Stimulus material from Study 3, translated from German to English. On the left, the guideline is shown, and on the right, the itineraries, which were presented in the standard way for all groups.

The stimulus material, procedure, etc., were similar to Studies 1 and 2, with the following exceptions: First, participants were informed that they needed to choose a mode of transport for a business trip^14^ that they would be taking with a senior colleague. Second, the effect of a *company guideline*^15^—which included a detailed explanation of the relatively low time savings of plane travel within Europe and emphasized the higher CO_2_ emissions associated with flying—was tested (see Figure 9). For this purpose, three groups (to which the participants were randomly assigned) were formed. The first group was first presented with the guideline and then had to choose a mode of transport. The second group, or its participants, first had to choose their mode of transport. They were then presented with the guideline. In a next step, they were then confronted again with their preference of mode of transport, which they could adjust as they wished. The last group (classic control group) did not receive a guideline; its participants only had to indicate a mode of transport preference (these three groups together form the independent variable: guideline). Third, in addition to plane and train, the bus was provided as a third mode of transport for the choice (dependent variable: preference). Fourth, participants had to answer guideline-related questions [each on a scale from 1 to 5 (1 = not at all, to 5 = very much)]. These questions concerned arguments mentioned in the guideline (e.g., “Within Europe, the time saved by flying is not that significant”; see “Argument ratings” section, also for the complete list of questions), or about the guideline itself (e.g., “It makes sense for a company to introduce such a guideline”; see “Guideline Acceptance and Compliance” section). Finally, participants had to indicate their perceived likelihood that they themselves and other employees would comply with the guideline, rated on a scale from 0 (not at all) to 100 (definitely).

### Results

Four participants that needed less than 2 or more than 11 min to complete the study were excluded from the analysis (these represented 2% of the total number of participants).

#### Mode of transport preference

**Figure 10:**
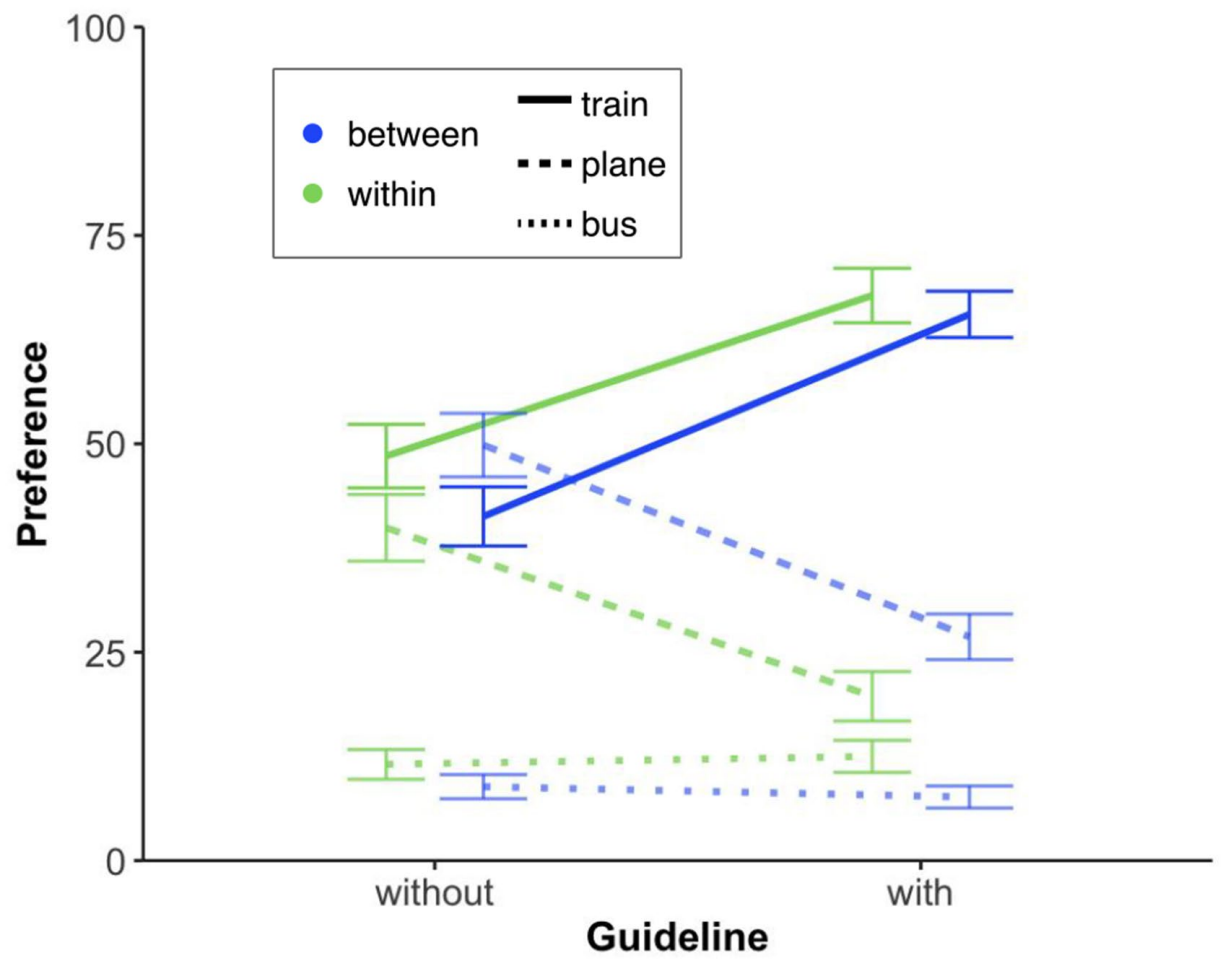
Mode of transport preferences with or without a guideline, showing the probability of choosing train, plane, or bus (summing to 100) on a 0–100 scale (0 = not an option, 100 = definitely preferred). Error bars represent standard errors of the mean.

Because the dependent variable—the distribution of 100 percentage points across plane, train, and bus—reflects a mutually dependent choice, we calculated a fractional multinomial logit model (see Papke and Wooldridge, 1996), choosing “plane” as the reference mode of transport (*N* = 266; log pseudo-likelihood = −237.25). Average marginal effects^16^ were calculated for each independent variable (guideline: without vs. with^17^; design: between vs. within^18^) on each mode of transport. For each mode of transport, the average marginal effect reflects the change from one level of the independent variable (e.g., guideline) compared to the other level of this variable chosen as the reference value. The results show that the presence of a guideline decreases the probability of using the plane by 22% (*p* < 0.001), increases the probability of using the train by 22% (*p* < 0.001), and has no effect on the probability of using the bus (*p* = 0.961). The design had no significant effect on either mode of transport (all other *p* > 0.054; see Figure 10).

#### Argument ratings

**Figure 11:**
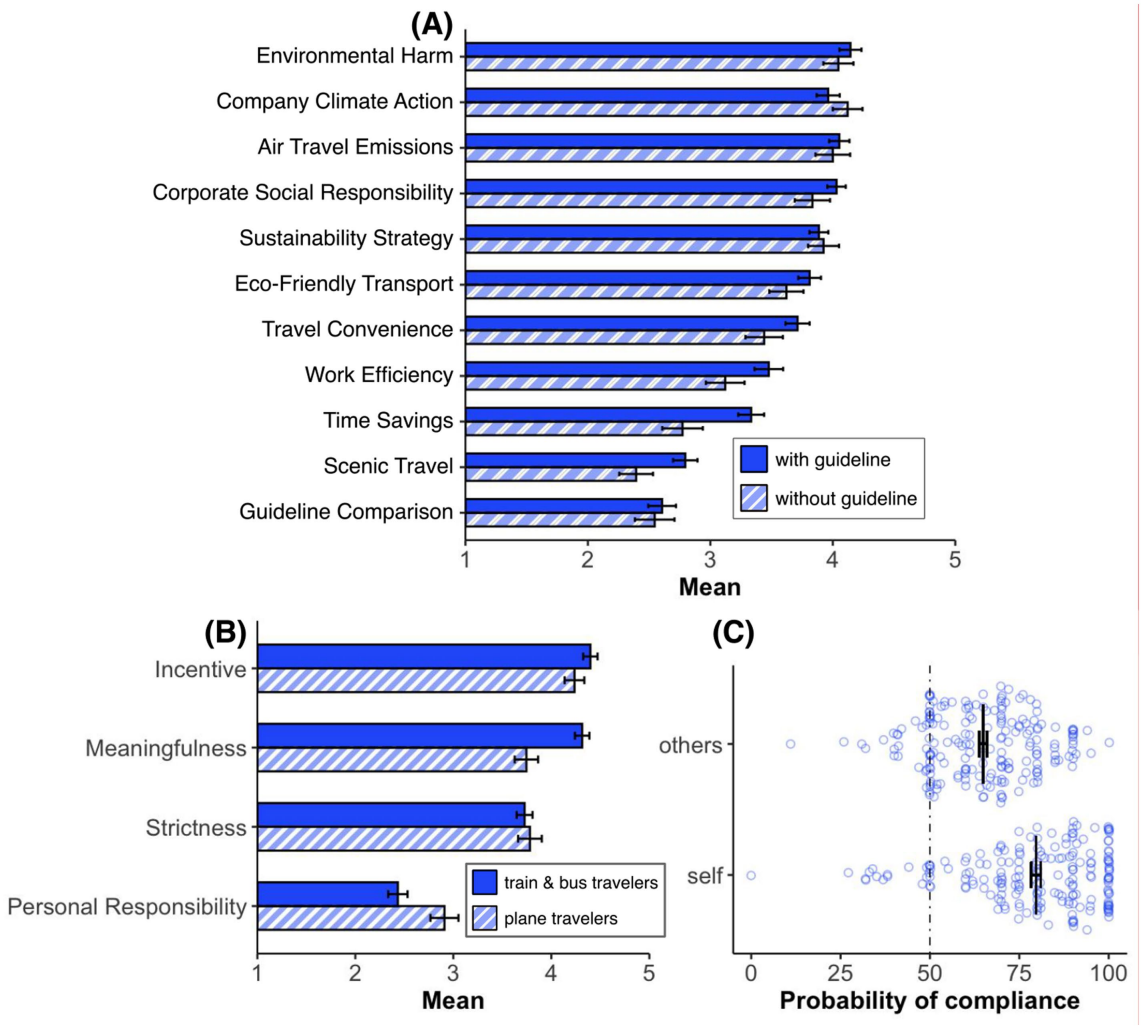
**(A)** Mean agreement ratings on a 5-point scale (1 = not convincing at all, 5 = very convincing) for arguments supporting the introduction of a company travel guideline. Translated from German to English: Environmental harm: “Flying is much more harmful to the environment than traveling by train or bus.” Company climate action: “The company is making a significant step towards climate neutrality.” Air travel emissions: “Air travel is responsible for 35% of the company’s greenhouse gas emissions.” Corporate social responsibility: “The company is acknowledging its social responsibility.” Sustainability strategy: “It is part of the company’s sustainability strategy.” Eco-friendly transport: “Traveling by train and bus are very environmentally friendly ways to travel.” Travel convenience: “For short distances, flying is often more cumbersome than traveling by train.” Work efficiency: “You can work more efficiently on a train than on an airplane.” Time savings: “Within Europe, the time saved by flying is not that significant.” Scenic travel: “On a train or bus, you can enjoy the landscape better.” Guideline comparison: “Many other comparable companies have introduced similar guidelines.” **(B)** Mean agreement levels on a 5-point scale (1 = “does not apply at all,” 5 = “completely applies”) for company travel guidelines among train & bus travelers versus plane travelers. The statements that were evaluated (translated from German to English) include Incentive: “Incentives should be created to fly less,” Meaningfulness: “It makes sense for a company to introduce such a guideline,” Strictness: “A non-binding guideline is not effective enough,” and Personal responsibility: “The decision to fly or not to fly should ultimately be left to the employees.” **(C)** Comparison of individuals’ expectations of their own compliance (self) versus their expectations of others’ compliance (others) with the travel guideline. Each point represents an individual response. Error bars represent standard errors of the mean.

To investigate whether the presentation of the guideline or not [independent variable: guideline (with, without)] influences the assessment of various arguments, such as environmental harm, differently, we conducted unpaired *t*-tests (Bonferroni-corrected) on all these arguments. We only found a significant effect of guideline regarding the argument “Within Europe, the time saved by flying is not as significant (abbr. Time Savings),” *t*(196) = −2.95, *p* = 0.04, *d* = −0.45; (all other *p*-values ≥0.22). The guideline thus made the participants aware that flying short distances does not really save time (see Figure 11A); just as the comprehensive travel itinerary presentation did (see Studies 1 and 2).

#### Guideline acceptance and compliance

Here we investigated, for example, guideline-related questions. Overall, the “meaningfulness” argument shows that participants appear to be in favor of the introduction of a company guide, although this agreement is significantly weaker among plane travelers (*n* = 55) than among train and bus travelers (*n* = 143), *t*(196) = −4.06, *p* < 0.001, *d* = −0.64. However, it is also clear that incentives are desired (see Figure 11B). Yet, it is still necessary to define what these should look like (e.g., travel time on the train could be regarded as working time). It can also be seen that plane travelers are significantly more likely to believe that the decision to fly or not should be their personal responsibility, *t*(196) = 2.60, *p* = 0.010, *d* = 0.41. Last but not least, participants rated themselves (M = 79.69) as more compliant with such a guideline, *t*(394) = 8.69, *p* < 0.001, *d* = 0.87, than others (M = 64.89; see Figure 11C).

### Discussion

Despite small differences in design, Study 3 shows that raising participants’ awareness of the fact that flying in Europe (i.e., for short distances) does not really save time has a similar effect on the choice of train, also expressed as a percentage, as in Studies 1 and 2, in which the comprehensive travel itinerary was presented. Accordingly, the goal of reducing business-related flight emissions can also be achieved with a simple guideline, even without coercion. However, possible intensifications should not be carelessly brushed aside. The difference in judging one’s own behavior compared to the behavior of others, in this case compliance to the mentioned guideline, is consistent with other psychological research showing that “most people strongly believe that they are just, virtuous, and moral, but view the average person as significantly less just” (Tappin and McKay, 2017, p. 1).

## Conclusion and discussion

We were able to show, that when implicitly known information such as “total travel time for flights also includes the time for security checks, etc.” is made explicit, the preference of *mode of transport* changes (i.e., fewer plane travels). Moreover, the results found were seen regardless of whether the information was made explicit, through the (visual) presentation of comprehensive travel itineraries (Studies 1 and 2) or through the simple (written) mention of a travel guideline (Study 3).

What underlying mechanism could explain the results? In this regard, we postulate *selective attention*, which brings implicit information into consciousness and thus enables its *semantic processing* (e.g., Treisman, 1964; Treisman et al., 1974). This allows “times for security checks, etc.” or more generally the attribute “additional time” to be considered in decision-making. Note that decision-making involves evaluating all relevant attributes (e.g., price, time effort) of the options (e.g., plane, train), weighing them up properly and then choosing the option with the highest expected value (EV). Hence, if attributes—as in the case of implicitly known attributes—are not attended, they are not considered when calculating the expected value of the option. Wright (1974) found that product evaluations and therefore product choice are often based on the few attributes that attract attention. Research in this area has furthermore shown that “the way people screen product information is related to the benefits they are seeking” (see Haley, 1971, p. 8). For example, consumers who want the benefit of caries prevention in a toothpaste pay particular attention to information about the product feature fluoride, while consumers who are looking for the taste of a toothpaste are more likely to look for features such as mint flavor (Haley, 1968). A similar observation can be made in Study 1, for example, regarding participants’ preference to travel by plane or train. When choosing the plane, time seems to be the decisive factor, while when choosing the train, convenience is the decisive factor (Ratneshwar et al., 1997). However, we also noted that such attention effects appear to be short-lived. The more time passes before a decision (plane or train) is made, the more the advantage of the formerly attended attribute disappears. The participants seem to have turned their attention to other attributes in the meantime, so that the attribute “additional time” is no longer included in the calculation of the EV. However, research still needs to be conducted into exactly *how* attention works. For example, findings from studies that have investigated how humor in advertising maintains attention could be helpful here (Goodrich et al., 2011).

In addition to a closer look at the role of attention, the question of whether information about attributes such as “additional time” may be deliberately avoided or ignored with regard to the decision must also be investigated. Although research on the topic of “information avoidance,” defined as “any behavior intended to prevent or delay the acquisition of available but potentially unwanted information” (Sweeny et al., 2010, p. 341), is still in its infancy, various authors (Sweeny et al., 2010; Gigerenzer and Garcia-Retamero, 2017; Golman et al., 2017) have shown its impact on people’s decisions. Thereby, information avoidance seems to be influenced by factors such as whether the information is a threat to one’s current beliefs or social norms (Hart et al., 2009; Foust and Taber, 2025). Since these factors are also important for choosing a more sustainable mode of transport, future research should investigate whether information avoidance also plays a role in scenarios like ours, and if so, what the driving factors are.

What might be potential study limitations? First, the content of the scenarios—a work-related conference trip as an employee of a medium-sized company. Such a work-related scenario could lead participants to be more sensitive to time efficiency, which—compared to a leisure travel scenario—could lead to a different response to the information on total travel time. Future studies should therefore investigate whether the observed results can also be generalized for other travel scenarios (e.g., leisure, visiting family). Second, the study sample which consisted primarily of university students residing in Switzerland—a cohort that is young, female and likely to be environmentally-friendly as in, for example, Meyer (2015); or Kirby and Zwickle (2021). This contrasts with the typical European business traveler (Cats, 2025; Schmalz et al., 2021), who, according to Cats (2025), is in his early 40s, male and less environmentally friendly. This is because the business traveler only takes the train for around a third of business trips in Europe. The fact that the nudge led to a substantial additional shift (i.e., toward the train)—even in Studies 1 and 2, where environmental issues were never mentioned—suggests that sudden awareness of the hidden time costs, rather than pre-existing environmentally-friendlier behavior, is responsible for the effect. However, future field studies with real business travelers and real booking data would be required to verify to what extent the effect exceeds the effect generated with our student sample. Third, hypothetical (via experiments) rather than actual travel preferences were measured. Although experimental results are often valid approximations of real-world behavior (Hainmueller et al., 2015; Mullinix et al., 2015; Stroud and Van Duyn, 2020), eliciting actual preferences can confirm the effectiveness of our manipulation in the real world. Fourth, the effect of price was only examined in scenarios where plane travel was more expensive than train travel. In this context, it was found that the effectiveness of the manipulation disappeared when flights were very cheap. Future research could also investigate scenarios where plane travel is cheaper than train travel. This could support the generalizability of the results of our manipulation. Lastly, the focus of our studies was on analyzing the impact of the itinerary presentation form on mode of transport. Although we also examined factors such as time and cost, our samples (although already quite large) would have been too small to include these factors in an extended analysis and examine their interaction with the itinerary presentation form on mode of transport. However, it would be interesting to investigate in future studies whether, for example, people for whom saving time is most important are more influenced by the indication of total travel time.

Before we end with a conclusion, we would like to point out the general significance of the results of these studies. Every day we make choices with *incomplete* information. For example, when we book a hotel room in Switzerland, we are told the price but not the tourist tax, which must be paid additionally, on arrival at the hotel. Even if the consequences in this example are less far-reaching, they are still important in other cases. For example, if we have to decide on the treatment of a serious illness, where doctors provide information on the success rate and side effects, but not, for example, on the recovery time. In both cases, this could mean that our choice would be different than if we had been explicitly provided also with the implicitly known information.

To conclude, our studies have shown that simple manipulations (e.g., changing the standard itinerary presentation form) have a major impact on people preferring a more environmentally friendly mode of transport. Hence, by implementing these insights (e.g., by booking platforms), an important contribution could be made to reducing CO_2_ emissions and thus the climate crisis.

## Data Availability

The datasets presented in this article are not readily available because only aggregated data is available upon request. Requests to access the datasets should be directed to catr@zhaw.ch.

## Footnotes

^1^Risky decisions such as in the Asian disease problem.

^2^As a reminder: all participants knew the starting and ending point of the trip. In addition, all participants were informed, for example, that the time from the Florence airport to the convention hall was approximately 1 h and 15 min. Therefore, participants in the standard condition also had a rough estimate of the door-to-door travel time.

^3^For conciseness we use the term preference (not choice) from now on.

^4^0–100 scales were used to assess, for example, preferences, intentions, etc. (see, e.g., Haase et al., 2013). A detailed justification for the use of this type of scale can be found in the Supplementary material.

^5^Question translated from German to English: *Specific travel factors*: “How would you rate the (plane/train) option in terms of the following dimensions? Please note that this refers to the entire journey from Zurich Main Station (Zürich HB) to the conference venue (Florence Campo di Marte).” (The dimensions were as follows: convenience, time effort, reliability); *General travel factors*: “How important were the following aspects when choosing your travel option?” (The aspects were: comfort, time effort, number of changes, etc.; see Figure 3B).

^6^Based on reaction time (RT) histograms.

^7^The detailed itinerary contained a slight error: the total travel time (4 h 12 min) did not match the sum of the individual trip sections (4 h 48 min). The mistake was fixed after about half the participants had already completed the study. We statistically checked and found no significant difference for the participants before and after the mistake was corrected.

^8^Decision time (in seconds), measured from the time participants were asked the question about their preferred mode of transportation to the time they responded. Furthermore, only participants with decision times greater than 0 s and less than 100 s were included in the analysis (*n* = 604).

^9^Note that participants had to answer the question regarding plane and train.

^10^If the travel option was assigned a value of below 50, it is coded as if the participant has chosen the plane; for values of 50 or more, as if he/she has chosen the train.

^11^Note, questions translated from German to English. General travel factors: “How important were the following aspects when choosing your travel option?” (The aspects were: reliability, number of changes, price, time effort, etc.; see Supplementary Table 1 or Supplementary Figure 1).

^12^Unless otherwise stated, both decisions, with and without price, were included in the model calculations, whereby this factor was treated as a within-subject one.

^13^Participants in the standard train itinerary condition were excluded from this analysis.

^14^This trip is a shorter journey, with the destination Cologne.

^15^Note, that only the standard itinerary presentation was used.

^16^The estimated *p*-values of the marginal effects were corrected using the Benjamini–Hochberg correction (Benjamini and Hochberg, 1995).

^17^Note, that the “guideline” level *with* is formed from the data of groups 1 and 2.

^18^Note, that the “design” level *between* is formed from the data of groups 1 and 3, while the “design” level *within* level is only formed from the data of group 2.
