# Supplementary material for: Changing tracks: how different visual presentations of travel itineraries impact the choice between plane and train
Source: Front Psychol. 2025 Jul 21;16:1588280. doi: 10.3389/fpsyg.2025.1588280 (PMC12320055; doi:10.3389/fpsyg.2025.1588280)
Supplement: Supplementary file 1 [file Table_1.DOCX]

Supplementary Material

# On continuous rating scales

The choice of the “0-100 (rating) scale” as a measurement method is an implementation of a visual analogue (or analog) scale (VAS). A VAS captures subjective assessments (such as pain or satisfaction) by having respondents mark a point on a *continuum* rather than choosing from *discrete* response options (as with, for example, Likert scales). This method, which typically presents a line anchored only by its extreme endpoints (e.g., 0 = “not at all” and 100 = “completely”), is recognized for its ability to provide interval (or continuous) data. Research shows that a 0-100 scale performs just as well as a *Likert scale* (discrete response options) in terms of psychometric criteria (e.g., reliability, validity; see, for example, Lewis and Erdinç, 2017) and, for our purposes, in measuring behavioral intentions (Haase et al., 2013). Furthermore, because a 0-100 scale captures more nuanced responses than forced-choice questions (e.g., plane or train; also applies to Likert scales), it can provide richer, more predictive data. For example, a 0-100 probability scale in public opinion research has maintained the predictive accuracy of forced-choice questions while halving the rate of “undecided” responses and revealing important nuances in voter certainty (Flannelly et al., 2000). This method of measurement is not new, it has a long history in psychology that began with the graphic rating scale over a century ago (Hayes and Patterson, 1921) and is continuously refined by modern methods that improve its properties (Lubiano et al., 2021). The 0-100 scale is therefore not only, for example, a psychometrically sound method, it is also considered more user-friendly (Lewis and Erdinç, 2017) and particularly suitable for internet-based research (see, for example, Reips & Funke, 2008).

# Supplementary Figures and Tables


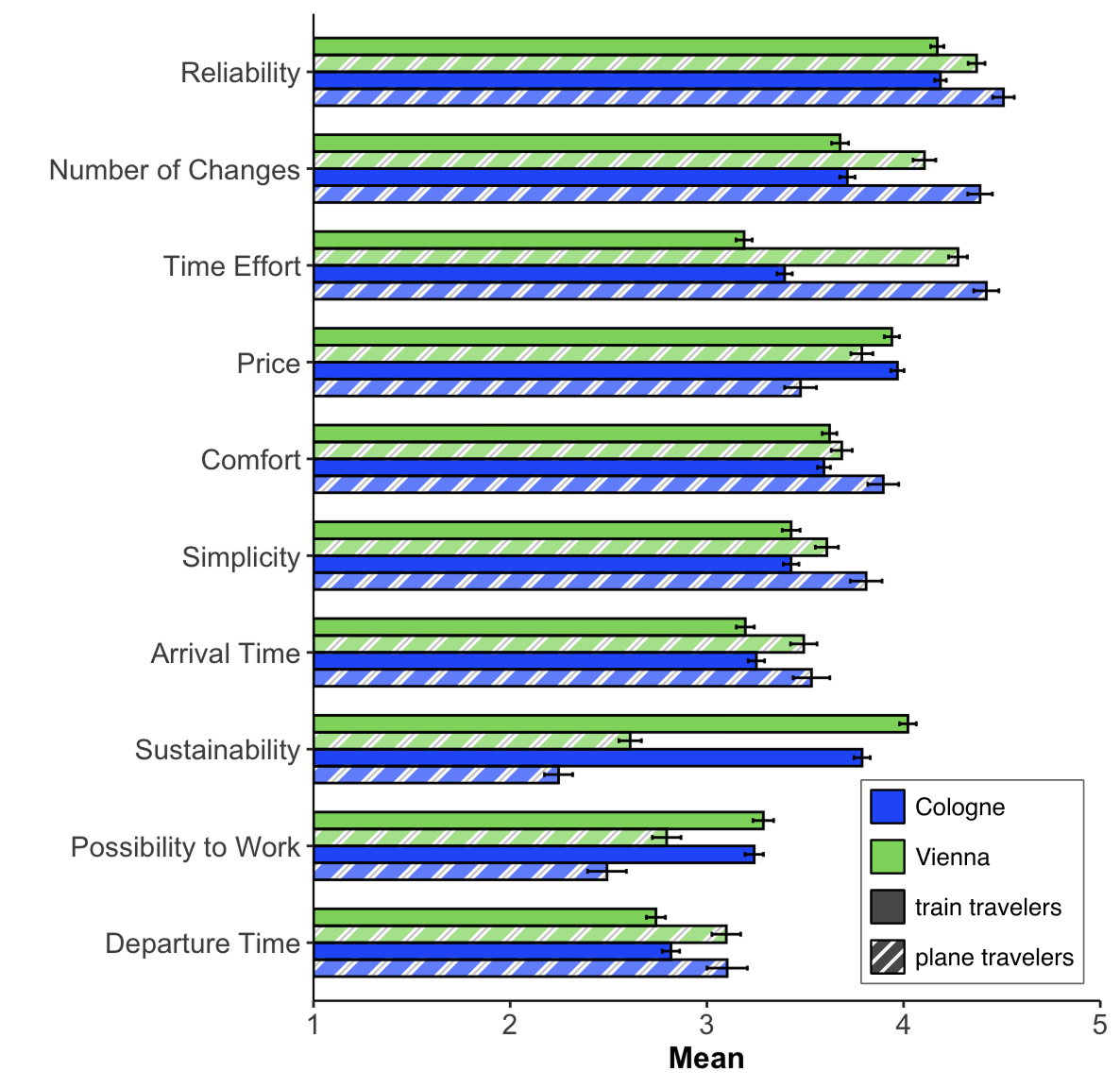


**Supplementary Figure 1.** Mean importance scores for general decision factors by destination, for plane and train travelers, rated from 1 (not important at all) to 5 (very important). Factors are ordered by their overall mean. Error bars represent standard errors of the mean.

**Supplementary Table 1.**

*T-Test Results*

|  | Cologne | |  | Vienna | | | |  |
| --- | --- | --- | --- | --- | --- | --- | --- | --- |
| General Travel Factor | t-value (df) | p | d | |  | t-value (df) | p | d |
| Reliability | -4.49 (764) | < .001 | -0.44 | |  | -3.58 (764) | .007 | -0.27 |
| Number of Changes | -7.30 (764) | < .001 | -0.71 | |  | -5.88 (764) | < .001 | -0.45 |
| Price | 5.85 (764) | < .001 | 0.57 | |  | 2.28 (764) | .462 | 0.17 |
| Time Effort | -11.11 (764) | < .001 | -1.08 | |  | -16.25 (764) | < .001 | -1.24 |
| Comfort | -3.69 (764) | .005 | -0.36 | |  | -0.97 (764) | 1 | -0.07 |
| Simplicity | -3.94 (764) | .002 | -0.38 | |  | -2.39 (764) | .34 | -0.18 |
| Arrival Time | -2.72 (764) | .133 | -0.27 | |  | -3.72 (764) | .004 | -0.28 |
| Sustainability | 15.67 (764) | < .001 | 1.53 | |  | 19.61 (764) | < .001 | 1.49 |
| Possibility to Work | 6.56 (764) | < .001 | 0.64 | |  | 5.47 (764) | < .001 | 0.42 |
| Departure Time | -2.59 (764) | .193 | -0.25 | |  | -4.19 (764) | < .001 | -0.32 |

**References**

Flannelly, K. J., Flannelly, L. T., and McLeod, C. C. (2000). Comparison of election predictions, voter certainty, and candidate choice on political polls. Psychol. Rep. 86, 755–763. doi: 10.2466/pr0.2000.86.3.755

Haase, N., Renkewitz, F., and Betsch, C. (2013). The measurement of subjective probability: evaluating the sensitivity and accuracy of various scales. Risk Anal. 33, 1812–1828. doi: 10.1111/risa.12025

Hayes, M. H., and Patterson, D. G. (1921). Experimental development of the graphic rating method. Psychol. Bull. 18, 98–99. doi: 10.1037/h0067885

Lewis, J. R., and Erdinç, O. (2017). User experience rating scales with 7, 11, or 101 points: does it matter? J. Usability Stud. 12, 73–91.

Lubiano, M. A., García-Izquierdo, A. L., Gil, M. Á., et al. (2021). Fuzzy rating scales: does internal consistency of a measurement scale benefit from coping with imprecision and individual differences? Inf. Sci. 550, 91–108. doi: 10.1016/j.ins.2020.10.021

Reips, U.-D., and Funke, F. (2008). Interval-level measurement with visual analogue scales in Internet based research: VAS Generator. Behav. Res. Methods 40, 699–704. doi: 10.3758/BRM.40.3.699
